# Consistent improvement with eculizumab across muscle groups in myasthenia gravis

**DOI:** 10.1002/acn3.51121

**Published:** 2020-07-22

**Authors:** Renato Mantegazza, Fanny L. O'Brien, Marcus Yountz, James F. Howard, for the REGAIN study group, Claudio Gabriel Mazia, Miguel Wilken, Fabio Barroso, Juliet Saba, Marcelo Rugiero, Mariela Bettini, Marcelo Chaves, Gonzalo Vidal, Alejandra Dalila Garcia, Jan De Bleecker, Guy Van den Abeele, Kathy de Koning, Katrien De Mey, Rudy Mercelis, Délphine Mahieu, Linda Wagemaekers, Philip Van Damme, Annelies Depreitere, Caroline Schotte, Charlotte Smetcoren, Olivier Stevens, Sien Van Daele, Nicolas Vandenbussche, Annelies Vanhee, Sarah Verjans, Jan Vynckier, Ann D’Hont, Petra Tilkin, Alzira Alves de Siqueira Carvalho, Igor Dias Brockhausen, David Feder, Daniel Ambrosio, Pamela César, Ana Paula Melo, Renata Martins Ribeiro, Rosana Rocha, Bruno Bezerra Rosa, Thabata Veiga, Luiz Augusto da Silva, Murilo Santos Engel, Jordana Gonçalves Geraldo, Maria da Penha Ananias Morita, Erica Nogueira Coelho, Gabriel Paiva, Marina Pozo, Natalia Prando, Debora Dada Martineli Torres, Cristiani Fernanda Butinhao, Gustavo Duran, Tomás Augusto Suriane Fialho, Tamires Cristina Gomes da Silva, Luiz Otavio Maia Gonçalves, Lucas Eduardo Pazetto, Luciana Renata Cubas Volpe, Luciana Souza Duca, Maurício André Gheller Friedrich, Alexandre Guerreiro, Henrique Mohr, Maurer Pereira Martins, Daiane da Cruz Pacheco, Luciana Ferreira, Ana Paula Macagnan, Graziela Pinto, Aline de Cassia Santos, Acary Souza Bulle Oliveira, Ana Carolina Amaral de Andrade, Marcelo Annes, Liene Duarte Silva, Valeria Cavalcante Lino, Wladimir Pinto, Natália Assis, Fernanda Carrara, Carolina Miranda, Iandra Souza, Patrícia Fernandes, Zaeem Siddiqi, Cecile Phan, Jeffrey Narayan, Derrick Blackmore, Ashley Mallon, Rikki Roderus, Elizabeth Watt, Stanislav Vohanka, Josef Bednarik, Magda Chmelikova, Marek Cierny, Stanislava Toncrova, Jana Junkerova, Barbora Kurkova, Katarina Reguliova, Olga Zapletalova, Jiri Pitha, Iveta Novakova, Michaela Tyblova, Ivana Jurajdova, Marcela Wolfova, Henning Andersen, Thomas Harbo, Lotte Vinge, Susanne Krogh, Anita Mogensen, John Vissing, Joan Højgaard, Nanna Witting, Anne Mette Ostergaard Autzen, Jane Pedersen, Juha‐Pekka Erälinna, Mikko Laaksonen, Olli Oksaranta, Tuula Harrison, Jaana Eriksson, Csilla Rozsa, Melinda Horvath, Gabor Lovas, Judit Matolcsi, Gyorgyi Szabo, Gedeonne Jakab, Brigitta Szabadosne, Laszlo Vecsei, Livia Dezsi, Edina Varga, Monika Konyane, Giovanni Antonini, Antonella Di Pasquale, Matteo Garibaldi, Stefania Morino, Fernanda Troili, Laura Fionda, Antonella Pasquale, Amelia Evoli, Paolo Emilio Alboini, Valentina D’Amato, Raffaele Iorio, Maurizio Inghilleri, Laura Fionda, Vittorio Frasca, Elena Giacomelli, Maria Gori, Diego Lopergolo, Emanuela Onesti, Vittorio Frasca, Maria Gabriele, Francesco Saccà, Alessandro Filla, Teresa Costabile, Enrico Marano, Angiola Fasanaro, Angela Marsili, Giorgia Puorro, Carlo Antozzi, Silvia Bonanno, Giorgia Camera, Alberta Locatelli, Lorenzo Maggi, Maria Pasanisi, Angela Campanella, Akiyuki Uzawa, Tetsuya Kanai, Naoki Kawaguchi, Masahiro Mori, Yoko Kaneko, Akiko Kanzaki, Eri Kobayashi, Hiroyuki Murai, Katsuhisa Masaki, Dai Matsuse, Takuya Matsushita, Taira Uehara, Misa Shimpo, Maki Jingu, Keiko Kikutake, Yumiko Nakamura, Yoshiko Sano, Kimiaki Utsugisawa, Yuriko Nagane, Ikuko Kamegamori, Tomoko Tsuda, Yuko Fujii, Kazumi Futono, Yukiko Ozawa, Aya Mizugami, Yuka Saito, Makoto Samukawa, Hidekazu Suzuki, Miyuki Morikawa, Sachiko Kamakura, Eriko Miyawaki, Meinoshin Okumura, Soichiro Funaka, Tomohiro Kawamura, Masayuki Nakamori, Masanori Takahashi, Namie Taichi, Tomoya Hasuike, Eriko Higuchi, Hisako Kobayashi, Kaori Osakada, Hirokazu Shiraishi, Teiichiro Miyazaki, Masakatsu Motomura, Akihiro Mukaino, Shunsuke Yoshimura, Shizuka Asada, Seiko Yoshida, Shoko Amamoto, Tomomi Kobashikawa, Megumi Koga, Yasuko Maeda, Kazumi Takada, Mihoko Takada, Masako Tsurumaru, Yumi Yamashita, Yasushi Suzuki, Tetsuya Akiyama, Koichi Narikawa, Ohito Tano, Kenichi Tsukita, Rikako Kurihara, Fumie Meguro, Yusuke Fukuda, Miwako Sato, Tomihiro Imai, Emiko Tsuda, Shun Shimohama, Takashi Hayashi, Shin Hisahara, Tomihiro Imai, Jun Kawamata, Takashi Murahara, Masaki Saitoh, Shun Shimohama, Shuichiro Suzuki, Daisuke Yamamoto, Yoko Ishiyama, Naoko Ishiyama, Mayuko Noshiro, Rumi Takeyama, Kaori Uwasa, Ikuko Yasuda, Anneke van der Kooi, Marianne de Visser, Tamar Gibson, Byung‐Jo Kim, Chang Nyoung Lee, Yong Seo Koo, Hung Youl Seok, Hoo Nam Kang, HyeJin Ra, Byoung Joon Kim, Eun Bin Cho, MiSong Choi, HyeLim Lee, Ju‐Hong Min, Jinmyoung Seok, JiEun Lee, Da Yoon Koh, JuYoung Kwon, SangAe Park, Eun Haw Choi, Yoon‐Ho Hong, So‐Hyun Ahn, Dae Lim Koo, Jae‐Sung Lim, Chae Won Shin, Ji Ye Hwang, Miri Kim, Seung Min Kim, Ha‐Neul Jeong, JinWoo Jung, Yool‐hee Kim, Hyung Seok Lee, Ha Young Shin, Eun Bi Hwang, Miju Shin, Carlos Casasnovas, Maria Antonia Alberti Aguilo, Christian Homedes‐Pedret, Natalia Julia Palacios, Laura Diez Porras, Valentina Velez Santamaria, Ana Lazaro, Josep Gamez Carbonell, Pilar Sune, Maria Salvado Figueras, Gisela Gili, Gonzalo Mazuela, Isabel Illa, Elena Cortes Vicente, Jordi Diaz‐Manera, Luis Antonio Querol Gutiérrez, Ricardo Rojas Garcia, Nuria Vidal, Elisabet Arribas‐Ibar, Exuperio Diez Tejedor, Pilar Gomez Salcedo, Mireya Fernandez‐Fournier, Pedro Lopez Ruiz, Francisco Javier Rodriguez de Rivera, Mireya Fernandez‐Fournier, Maria Sastre, Fredrik Piehl, Albert Hietala, Lena Bjarbo, Ihsan Sengun, Arzu Meherremova, Pinar Ozcelik, Bengu Balkan, Celal Tuga, Muzeyyen Ugur, Sevim Erdem‐Ozdamar, Can Ebru Bekircan‐Kurt, Nazire Pinar Acar, Ezgi Yilmaz, Yagmur Caliskan, Gulsah Orsel, Husnu Efendi, Seda Aydinlik, Hakan Cavus, Ayse Kutlu, Gulsah Becerikli, Cansu Semiz, Ozlem Tun, Murat Terzi, Baki Dogan, Musa Kazim Onar, Sedat Sen, Tugce Kirbas Cavdar, Adife Veske, Fiona Norwood, Aikaterini Dimitriou, Jakit Gollogly, Mohamed Mahdi‐Rogers, Arshira Seddigh, Giannis Sokratous, Gal Maier, Faisal Sohail, Saiju Jacob, Girija Sadalage, Pravin Torane, Claire Brown, Amna Shah, Sivakumar Sathasivam, Heike Arndt, Debbie Davies, Dave Watling, Anthony Amato, Thomas Cochrane, Mohammed Salajegheh, Kristen Roe, Katherine Amato, Shirli Toska, Gil Wolfe, Nicholas Silvestri, Kara Patrick, Karen Zakalik, Jonathan Katz, Robert Miller, Marguerite Engel, Dallas Forshew, Elena Bravver, Benjamin Brooks, Mohammed Sanjak, Sarah Plevka, Maryanne Burdette, Scott Cunningham, Megan Kramer, Joanne Nemeth, Clara Schommer, Tierney Scott, Vern Juel, Jeffrey Guptill, Lisa Hobson‐Webb, Janice Massey, Kate Beck, Donna Carnes, John Loor, Amanda Anderson, Robert Pascuzzi, Cynthia Bodkin, John Kincaid, Riley Snook, Sandra Guingrich, Angela Micheels, Vinay Chaudhry, Andrea Corse, Betsy Mosmiller, Andrea Kelley, Doreen Ho, Jayashri Srinivasan, Michal Vytopil, Jordan Jara, Nicholas Ventura, Cynthia Carter, Craig Donahue, Carol Herbert, Stephanie Scala, Elaine Weiner, Sharmeen Alam, Jonathan McKinnon, Laura Haar, Naya McKinnon, Karan Alcon, Kaitlyn McKenna, Nadia Sattar, Kevin Daniels, Dennis Jeffery, Miriam Freimer, Joseph Chad Hoyle, John Kissel, Julie Agriesti, Sharon Chelnick, Louisa Mezache, Colleen Pineda, Filiz Muharrem, Chafic Karam, Julie Khoury, Tessa Marburger, Harpreet Kaur, Diana Dimitrova, James Gilchrist, Brajesh Agrawal, Mona Elsayed, Stephanie Kohlrus, Angela Ardoin, Taylor Darnell, Laura Golden, Barbara Lokaitis, Jenna Seelbach, Srikanth Muppidi, Neelam Goyal, Sarada Sakamuri, Yuen T So, Shirley Paulose, Sabrina Pol, Lesly Welsh, Ratna Bhavaraju‐Sanka, Alejandro Tobon Gonzalez, Lorraine Dishman, Floyd Jones, Anna Gonzalez, Patricia Padilla, Amy Saklad, Marcela Silva, Sharon Nations, Jaya Trivedi, Steve Hopkins, Mohamed Kazamel, Mohammad Alsharabati, Liang Lu, Kenkichi Nozaki, Sandi Mumfrey‐Thomas, Amy Woodall, Tahseen Mozaffar, Tiyonnoh Cash, Namita Goyal, Gulmohor Roy, Veena Mathew, Fatima Maqsood, Brian Minton, H James Jones, Jeffrey Rosenfeld, Rebekah Garcia, Laura Echevarria, Sonia Garcia, Michael Pulley, Shachie Aranke, Alan Ross Berger, Jaimin Shah, Yasmeen Shabbir, Lisa Smith, Mary Varghese, Yasmeen Shabbir, Laurie Gutmann, Ludwig Gutmann, Nivedita Jerath, Christopher Nance, Andrea Swenson, Heena Olalde, Nicole Kressin, Jeri Sieren, Richard Barohn, Mazen Dimachkie, Melanie Glenn, April McVey, Mamatha Pasnoor, Jeffery Statland, Yunxia Wang, Tina Liu, Kelley Emmons, Nicole Jenci, Jerry Locheke, Alex Fondaw, Kathryn Johns, Gabrielle Rico, Maureen Walsh, Laura Herbelin, Charlene Hafer‐Macko, Justin Kwan, Lindsay Zilliox, Karen Callison, Valerie Young, Beth DiSanzo, Kerry Naunton, Michael Benatar, Martin Bilsker, Khema Sharma, Anne Cooley, Eliana Reyes, Sara‐Claude Michon, Danielle Sheldon, Julie Steele, Chafic Karam, Rebecca Traub, Manisha Chopra, Tuan Vu, Lara Katzin, Terry McClain, Brittany Harvey, Adam Hart, Kristin Huynh, Said Beydoun, Amaiak Chilingaryan, Victor Doan, Brian Droker, Hui Gong, Sanaz Karimi, Frank Lin, Terry McClain, Krishna Polaka, Akshay Shah, Anh Tran, Salma Akhter, Ali Malekniazi, Rup Tandan, Michael Hehir, Waqar Waheed, Shannon Lucy, Michael Weiss, Jane Distad, Susan Strom, Sharon Downing, Bryan Kim, Tulio Bertorini, Thomas Arnold, Kendrick Henderson, Rekha Pillai, Ye Liu, Lauren Wheeler, Jasmine Hewlett, Mollie Vanderhook, Richard Nowak, Daniel Dicapua, Benison Keung, Aditya Kumar, Huned Patwa, Kimberly Robeson, Irene Yang, Joan Nye, Hong Vu

**Affiliations:** ^1^ Neuroimmunology and Neuromuscular Diseases Unit Fondazione IRCCS Istituto Neurologico Carlo Besta Milan Italy; ^2^ Alexion Pharmaceuticals Boston MA; ^3^ Department of Neurology University of North Carolina Chapel Hill NC

## Abstract

**Objective:**

To assess whether eculizumab, a terminal complement inhibitor, improves patient‐ and physician‐reported outcomes (evaluated using the myasthenia gravis activities of daily living profile and the quantitative myasthenia gravis scale, respectively) in patients with refractory anti‐acetylcholine receptor antibody‐positive generalized myasthenia gravis across four domains, representing ocular, bulbar, respiratory, and limb/gross motor muscle groups.

**Methods:**

Patients with refractory anti‐acetylcholine receptor antibody‐positive generalized myasthenia gravis were randomized 1:1 to receive either placebo or eculizumab during the REGAIN study (NCT01997229). Patients who completed REGAIN were eligible to continue into the open‐label extension trial (NCT02301624) for up to 4 years. The four domain scores of each of the myasthenia gravis activities of daily living profile and the quantitative myasthenia gravis scale recorded throughout REGAIN and through 130 weeks of the open‐label extension were analyzed.

**Results:**

Of the 125 patients who participated in REGAIN, 117 enrolled in the open‐label extension; 61 had received placebo and 56 had received eculizumab during REGAIN. Patients experienced rapid improvements in total scores and all four domain scores of both the myasthenia gravis activities of daily living profile and the quantitative myasthenia gravis scale with eculizumab treatment. These improvements were sustained through 130 weeks of the open‐label extension.

**Interpretation:**

Eculizumab treatment elicits rapid and sustained improvements in muscle strength across ocular, bulbar, respiratory, and limb/gross motor muscle groups and in associated daily activities in patients with refractory anti‐acetylcholine receptor antibody‐positive generalized myasthenia gravis.

## Introduction

Myasthenia gravis (MG) is a rare autoimmune disorder characterized by fluctuating muscle weakness that carries a heavy disease burden and impairs quality of life.^1^ MG is mediated by autoantibodies that target the nicotinic acetylcholine receptor (AChR) and activate the complement cascade in over 70% of patients, resulting in structural damage to the neuromuscular junction.^2^, ^3^, ^4^, ^5^, ^6^, ^7^ This impairs neuromuscular transmission and contributes to muscle weakness, which eventually affects the face, neck, hands, and/or limbs in 70–80% of patients with MG (generalized MG [gMG]).^8^, ^9^ MG is typically managed with the use of immunosuppressive therapies (ISTs), but 10–15% of patients are considered to have refractory disease owing to intolerable IST‐related adverse events, inability to lower IST dose without clinical relapse, or requirement for regular maintenance intravenous immunoglobulin (IVIg) or plasma exchange treatment.^1^, ^10^, ^11^, ^12^, ^13^, ^14^ Persistent MG symptoms can adversely impact breathing, talking, swallowing, walking, and other activities requiring limb strength.^15^

Eculizumab (Soliris, Alexion Pharmaceuticals, Boston, MA, USA) is a humanized monoclonal antibody that binds with high affinity to the terminal complement protein C5. This inhibits enzymatic cleavage of C5 and prevents downstream signaling of both C5a‐induced chemotaxis of proinflammatory cells and formation of the C5b‐induced membrane attack complex,^16^ which is a primary driver of membrane damage at the neuromuscular junction in anti‐AChR antibody‐positive (AChR+) gMG.^17^, ^18^

Eculizumab has been shown to be well tolerated and to improve clinical outcomes in patients with refractory AChR+ gMG in the 6‐month, phase 3, randomized, double‐blind, placebo‐controlled REGAIN study (NCT01997229).^15^ Long‐term tolerability and improvements to clinical outcomes have also been shown in an interim analysis of the REGAIN open‐label extension (OLE; NCT02301624).^19^

The MG activities of daily living profile (MG‐ADL)^20^ and the quantitative MG scale (QMG)^21^ are validated, MG‐specific outcome measures, each of which comprise four domains representing ocular, bulbar, respiratory, and limb/gross motor muscle groups. The MG‐ADL and QMG were key, prospectively defined efficacy measures used to assess patient outcomes in REGAIN and the OLE. Both MG‐ADL and QMG mean total scores improved with eculizumab treatment during REGAIN and during long‐term follow‐up in the OLE trial.^15^, ^19^

The objective of this analysis was to evaluate the MG‐ADL and QMG domain scores for each muscle group in patients during REGAIN and its OLE to determine whether eculizumab is clinically beneficial across all muscle groups in patients with refractory AChR+ gMG. Our hypothesis was that eculizumab would elicit rapid and sustained improvements across all MG‐ADL and QMG domains.

## Methods

### Study design and participants

REGAIN (NCT01997229) was a 6‐month, phase 3, randomized, double‐blind, placebo‐controlled study of eculizumab in adults with refractory AChR+ gMG, for which full methodology has been published previously.^15^

Briefly, eligible patients were 18 years of age or older, had confirmed gMG with positive serology for AChR autoantibodies, had an MG‐ADL total score of 6 or greater, and had received two or more ISTs, or at least one IST with requirement for chronic IVIg or plasma exchange therapy, in the preceding 12 months. Full eligibility criteria have been published previously.^15^

Patients who chose to continue into the OLE (NCT02301624) were required to enter it within 2 weeks of completing REGAIN.^19^ All patients provided written, informed consent. Written approval for the study protocol and all study amendments was obtained from independent ethics committees or institutional review boards at all participating sites.

### Dosing

During REGAIN, participants were randomized to receive intravenous eculizumab (maintenance dose, 1200mg every 2 weeks following a 4‐week induction period) or matched placebo for 26 weeks.^15^

During the OLE, after a 4‐week blinded induction phase, all participants received open‐label eculizumab 1200 mg every 2 weeks for up to 4 years.^19^ Patients who had received eculizumab during REGAIN constituted the eculizumab/eculizumab group, and patients who had received placebo during REGAIN constituted the placebo/eculizumab group.

### Assessments

The MG‐ADL is a patient‐reported, 8‐item questionnaire that reports on the functional impact of muscle weakness on activities of daily living in patients with gMG. It comprises four domains, representing ocular (two items), bulbar (three items), respiratory (one item), and limb (two items) muscle groups, which assess visual, oral, breathing, and limb motor abilities, respectively.^20^ Each item is scored from 0 to 3, with a maximum total score of 24. The QMG is an objective, physician‐reported, 13‐item measure of muscle strength that comprises four domains, representing ocular (three ocular and facial muscle items), bulbar (two swallowing and speech items), respiratory (one forced vital capacity item), and gross motor (seven limb and axial motor items) muscle groups.^21^ Each item is scored from 0 to 3, with a maximum total score of 39. The scores for all domains of the MG‐ADL and QMG were recorded throughout REGAIN and its OLE. Assessments were performed weekly from week 1 to week 3 and then at weeks 4, 8, 12, 16, 20, 26, 40, and 52 in year 1, then every 6 months afterward and at each patient’s end‐of‐study visit. The MG‐ADL and QMG total and domain scores from REGAIN and the completed OLE are reported here.

Patients with an abnormal score (>0) at REGAIN baseline for a domain of either measure were included in the analysis of that domain for its respective measure. MG‐ADL and QMG mean total and mean domain scores were calculated for both the eculizumab and the placebo groups in the REGAIN study and for the eculizumab/eculizumab and placebo/eculizumab groups in the OLE.

### Statistical analysis

Two baselines were used for these analyses; the REGAIN baseline (REGAIN day 1) was used to allow for assessment of response to eculizumab from the start of REGAIN, and the open‐label baseline (the last available assessment before the first eculizumab dose in the OLE) was used to allow for assessment of response to eculizumab in the placebo/eculizumab group and assessment of maintenance of the response observed during REGAIN in the eculizumab/eculizumab group.^19^ Changes from REGAIN baseline to OLE week 130 (156 weeks, in total, for patients who had received eculizumab during REGAIN) and from open‐label baseline to OLE week 130 were evaluated. In addition, for the correlation analysis, eculizumab baseline was defined as the first dose of eculizumab received.

Repeated‐measures analyses for changes in MG‐ADL and QMG total and domain scores from open‐label baseline were performed. These data are presented as least‐squares means and 95% confidence intervals (CIs). Pearson’s correlation coefficients (*R*) by treatment group were determined for MG‐ADL and QMG total score changes from eculizumab baseline to last OLE assessment and for MG‐ADL and QMG total scores at last OLE assessment; thresholds for moderate and strong correlations were 0.4 and 0.6, respectively.^22^ All statistical analyses were performed using SAS version 9.4 (SAS Institute, Cary, NC, USA). This study did not have a data‐monitoring committee.

## Results

### Patient disposition

**Figure 1:**
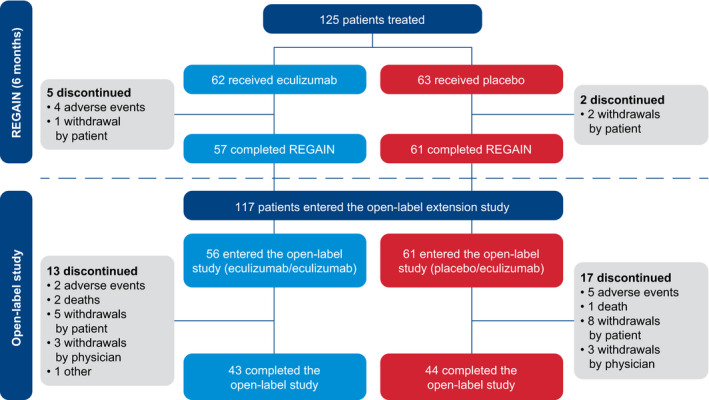
Patient disposition through January 2019. In total, 117 patients were enrolled in the OLE; 56 had received eculizumab and 61 had received placebo during REGAIN. At OLE study end, 30 patients in total had discontinued (23.2% of the eculizumab/eculizumab group [13/56] and 27.9% of the placebo/eculizumab group [17/61]), and 87 patients had completed the study (76.8% of the eculizumab/eculizumab group [43/56] and 72.1% of the placebo/eculizumab group [44/61]). OLE = open‐label extension. Figure reproduced with permission from Vissing J, Jacob S, Fujita KP, et al. ‘Minimal symptom expression’ in patients with acetylcholine receptor antibody‐positive refractory generalized myasthenia gravis treated with eculizumab. J Neurol 2020. (https://creativecommons.org/licenses/by/4.0/).

During REGAIN, 62 patients received eculizumab and 63 received placebo. Of the 118 patients who completed REGAIN, 117 (eculizumab/eculizumab, 56; placebo/eculizumab, 61) enrolled in the OLE (93.6% of REGAIN participants [117/125] entered the OLE). Almost three‐quarters (74.4%) of OLE participants (87/117) completed the OLE. Of the 30 patients who discontinued during the OLE, seven discontinued due to adverse events, and there were three deaths reported in patients with important comorbidities^19^ (Fig. 1). The median duration of eculizumab treatment during the OLE (from open‐label baseline to last OLE assessment) was 2.7 years (138.9 weeks; range, 0.1–196.0 weeks). Full final safety data will be published (Mantegazza R, et al. 2020. Minimal manifestations in eculizumab‐treated patients with refractory myasthenia gravis. Manuscript submitted for publication).

All OLE participants had at least one abnormal domain score for both MG‐ADL and QMG at REGAIN baseline and were, therefore, included in the analysis of total scores. The numbers of patients in the eculizumab/eculizumab and placebo/eculizumab groups, respectively, who had abnormal REGAIN baseline scores and were included in this analysis were: for the MG‐ADL domains, 55 and 59 for ocular, 54 and 55 for bulbar, 48 and 45 for respiratory, and 52 and 55 for limb; and for the QMG domains, 56 and 60 for ocular, 31 and 28 for bulbar, 28 and 36 for respiratory, and 56 and 61 for gross motor.

### MG‐ADL and QMG mean total scores

**Figure 2:**
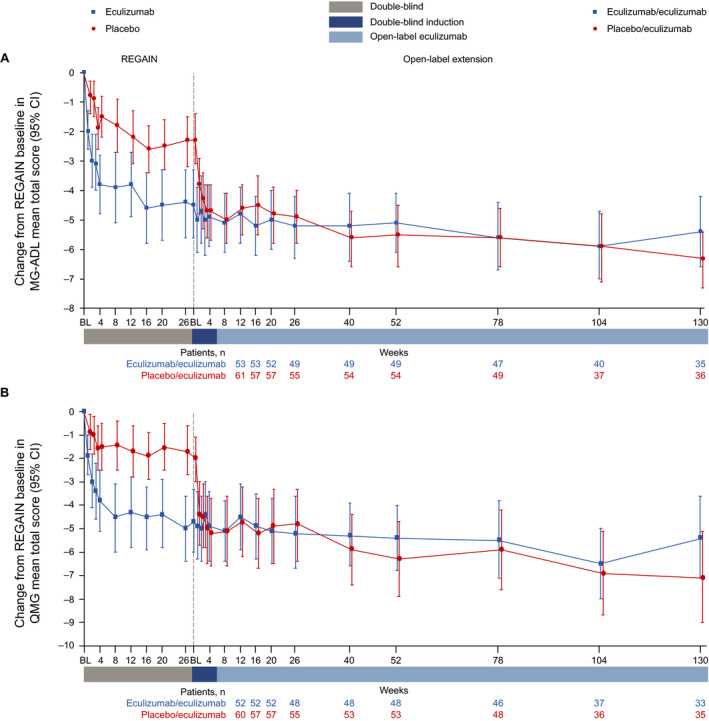
Changes in (A) MG‐ADL mean total score and (B) QMG mean total score from REGAIN baseline to OLE week 130. Patient numbers were not the same for each assessment. BL = baseline; CI = confidence interval; MG‐ADL = myasthenia gravis activities of daily living profile; OLE = open‐label extension; QMG = quantitative myasthenia gravis scale.

**Figure 3:**
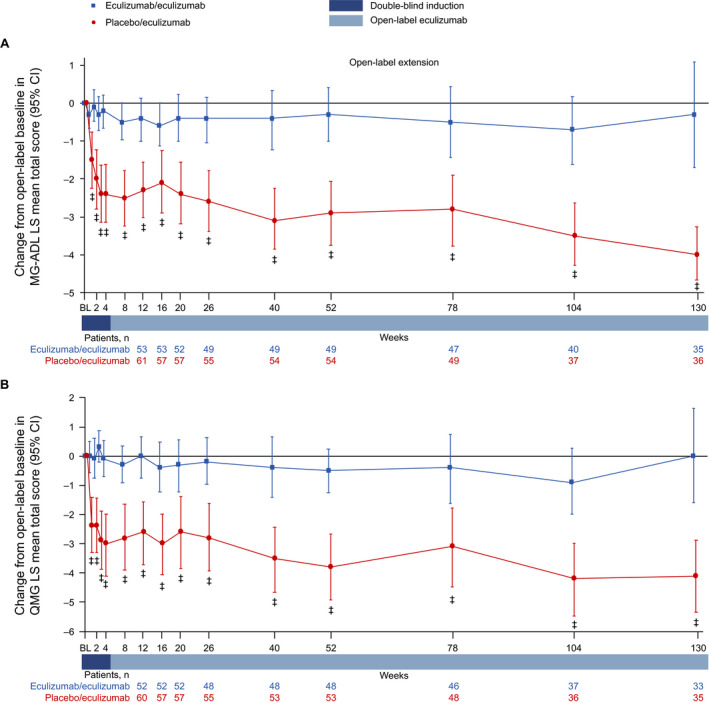
Changes in (A) MG‐ADL LS mean total score and (B) QMG LS mean total score from open‐label baseline to week 130. ^‡^ *P* ≤ 0.001 compared with open‐label extension baseline, repeated‐measures analysis. Patient numbers were not the same for each assessment. Stable scores in the eculizumab/eculizumab group are evidence of maintained improvement achieved during eculizumab treatment in REGAIN in these patients. BL = baseline; CI = confidence interval; LS = least‐squares; MG‐ADL = myasthenia gravis activities of daily living profile; QMG = quantitative myasthenia gravis scale.

For patients in the eculizumab/eculizumab group, the improvements achieved in both MG‐ADL and QMG mean total scores during REGAIN^15^ and the interim analysis of OLE data^19^ were found to be sustained in this final analysis of the complete OLE data through 130 weeks (Figs. 2, 3).

Patients in the placebo/eculizumab group experienced rapid improvements in MG‐ADL and QMG mean total scores from the start of eculizumab treatment in the OLE during the 4‐week blinded induction period (Figs. 2, 3). In this group, MG‐ADL and QMG mean total scores were significantly improved from open‐label baseline as early as OLE week 1 (*P ≤ *0.001), and these improvements remained significant for every week through 130 weeks (Fig. 3).

### MG‐ADL and QMG total score correlation

**Figure 4:**
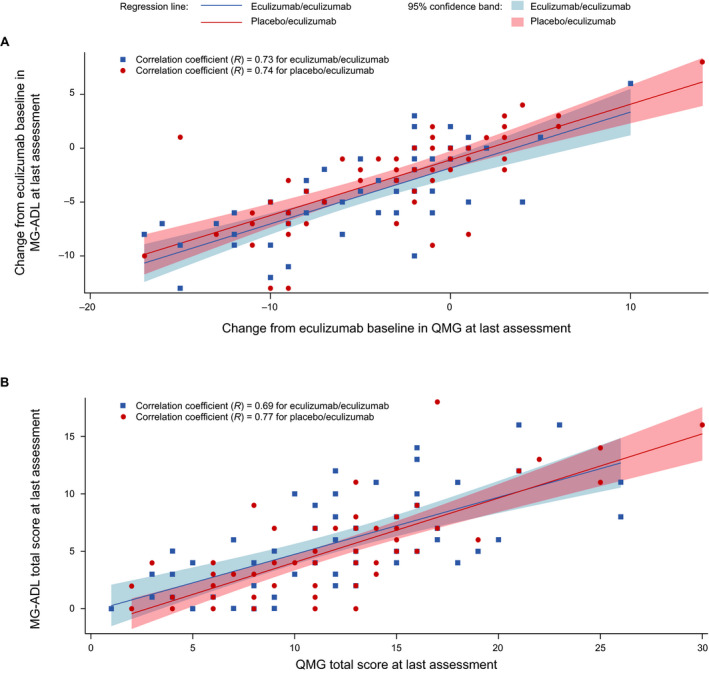
Correlations between MG‐ADL and QMG total scores (A) for changes from eculizumab baseline to last OLE assessment and (B) at last OLE assessment. Pearson’s correlation coefficients (*R*) were calculated for each treatment group. Each regression line was determined by a simple linear regression model of (A) change in MG‐ADL total score from REGAIN baseline to last OLE assessment against change in QMG total score from eculizumab baseline to last OLE assessment or (B) MG‐ADL total score at last OLE assessment as the response variable against QMG total score at last OLE assessment as the predictor variable for respective treatment groups, and its 95% confidence band was determined by the pointwise 95% confidence band. MG‐ADL = myasthenia gravis activities of daily living profile; OLE = open‐label extension; QMG = quantitative myasthenia gravis scale.

In the eculizumab/eculizumab group, there were strong correlations between changes in MG‐ADL and QMG total scores from eculizumab baseline to last OLE assessment (*R* = 0.73; 95% CI, 0.57–0.83; Fig. 4A) and between MG‐ADL and QMG total scores at last OLE assessment (*R* = 0.69; 95% CI, 0.51−0.80; Fig. 4B).

In the placebo/eculizumab group, the correlations between MG‐ADL and QMG total scores were also strong for changes in total scores from eculizumab baseline to last OLE assessment (*R* = 0.74, 95% CI, 0.59–0.83; Fig. 4A) and for total scores at last OLE assessment (*R* = 0.77; 95% CI, 0.64−0.86; Fig. 4B).

For the entire OLE population, the coefficients for the correlations between changes in MG‐ADL and QMG total scores from eculizumab baseline to last OLE assessment and between the total scores at last OLE assessment were 0.74 (95% CI, 0.65–0.81) and 0.73 (95% CI, 0.63–0.80), respectively.

### MG‐ADL domain scores

**Figure 5:**
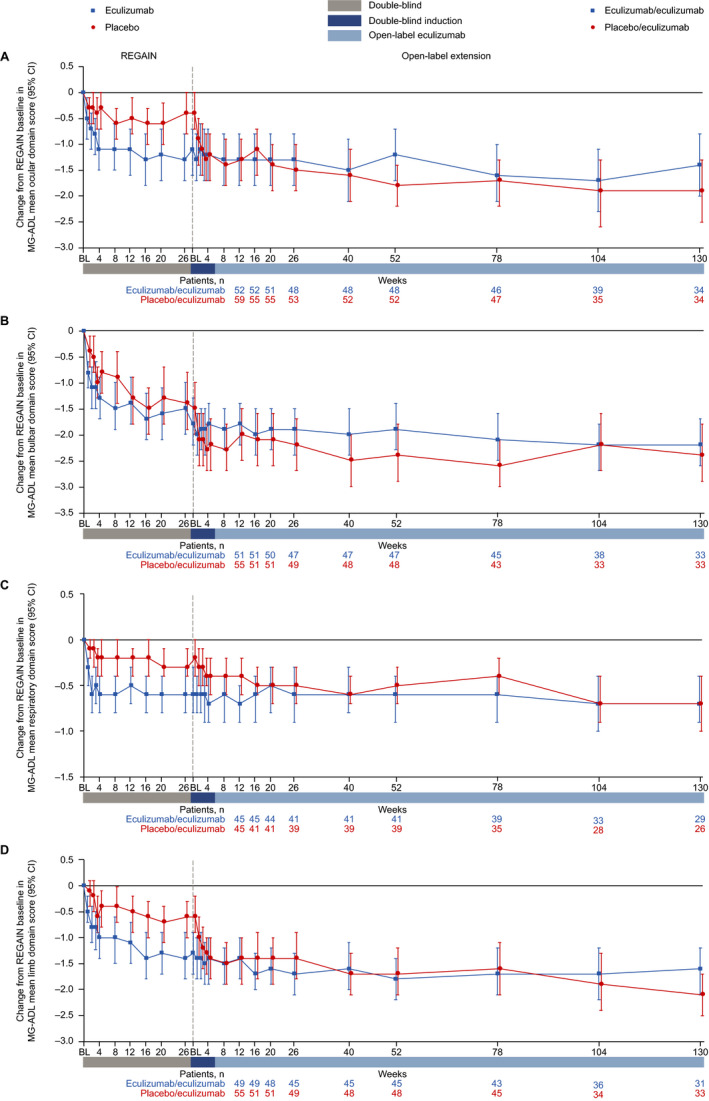
Changes in MG‐ADL mean domain scores from REGAIN baseline to OLE week 130 for (A) ocular, (B) bulbar, (C) respiratory, and (D) limb domains. Patient numbers were not the same for each assessment. Data are from patients with abnormal domain scores at REGAIN baseline only. BL = baseline; CI = confidence interval; MG‐ADL = myasthenia gravis activities of daily living profile; OLE = open‐label extension.

**Figure 6:**
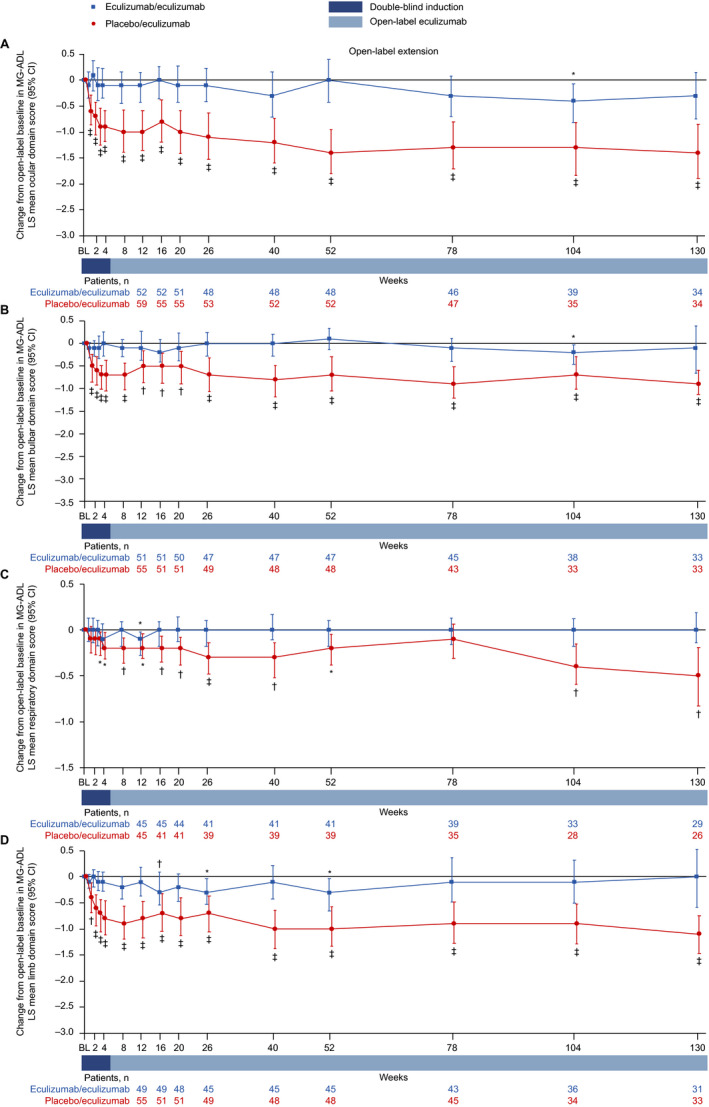
Changes in MG‐ADL LS mean domain scores from open‐label baseline to week 130 for (A) ocular, (B) bulbar, (C) respiratory, and (D) limb domains. *P ≤ 0.05, ^†^ *P* ≤ 0.01, ^‡^ *P* ≤ 0.001 compared with open‐label baseline, repeated‐measures analysis. Patient numbers were not the same for each assessment. Stable scores in the eculizumab/eculizumab group are evidence of maintained improvement achieved during eculizumab treatment in REGAIN in these patients. Data are from patients with abnormal domain scores at REGAIN baseline only. BL = baseline; CI = confidence interval; LS = least‐squares; MG‐ADL = myasthenia gravis activities of daily living profile.

Patients in the eculizumab/eculizumab group experienced rapid improvements across all four MG‐ADL domains during REGAIN, and this treatment effect was sustained through 130 weeks of the OLE (Fig. 5A–D and Fig. 6A–D). During REGAIN, peak improvements were observed by week 16 in all domains except the respiratory domain, in which it was observed by week 4 (Fig. 5A–D). Thereafter, the peak improvements achieved in REGAIN were sustained or increased in all domains during the OLE through 130 weeks (Fig. 6A–D).

In the placebo/eculizumab group, statistically significant improvements in scores from open‐label baseline were observed in the ocular, bulbar, and limb domains as early as week 1 and remained significant at each time point through 130 weeks (Fig. 6A, B, D). In the respiratory domain, the improvement in score was statistically significant by week 4 and remained significant at each time point through 130 weeks except at week 78 (Fig. 6C).

### QMG domain scores

**Figure 7:**
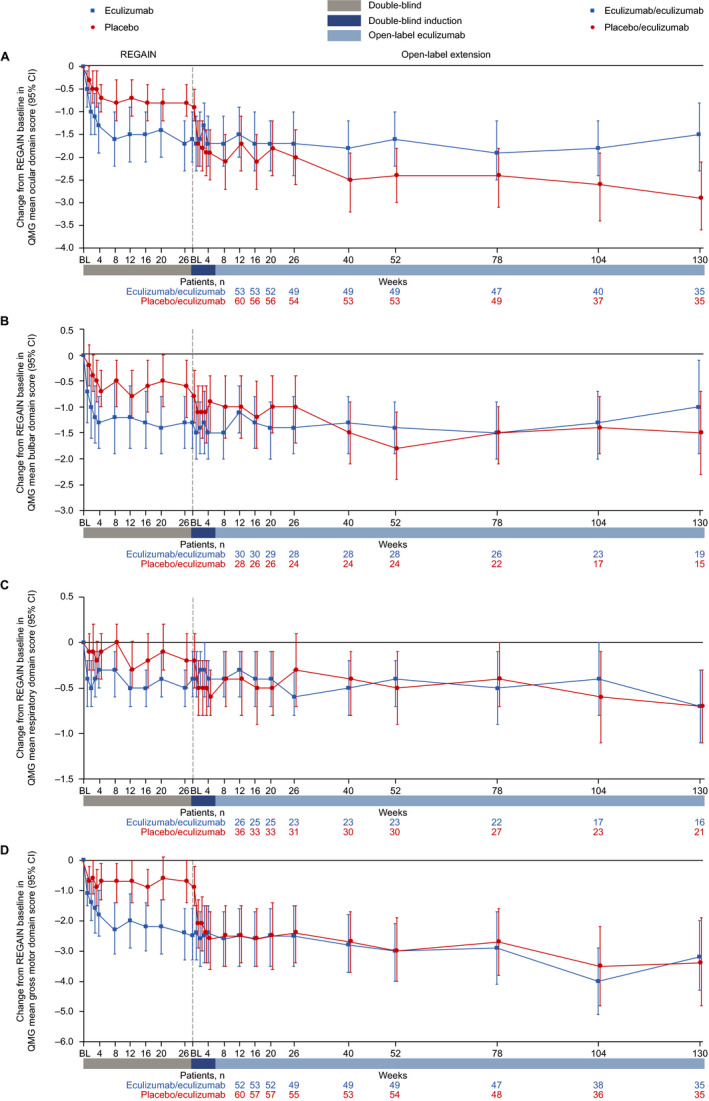
Changes in QMG mean domain scores from REGAIN baseline to OLE week 130 for (A) ocular, (B) bulbar, (C) respiratory, and (D) gross motor domains. Patient numbers were not the same for each assessment. Data are from patients with abnormal domain scores at REGAIN baseline only. BL = baseline; CI = confidence interval; OLE = open‐label extension; QMG = quantitative myasthenia gravis scale.

**Figure 8:**
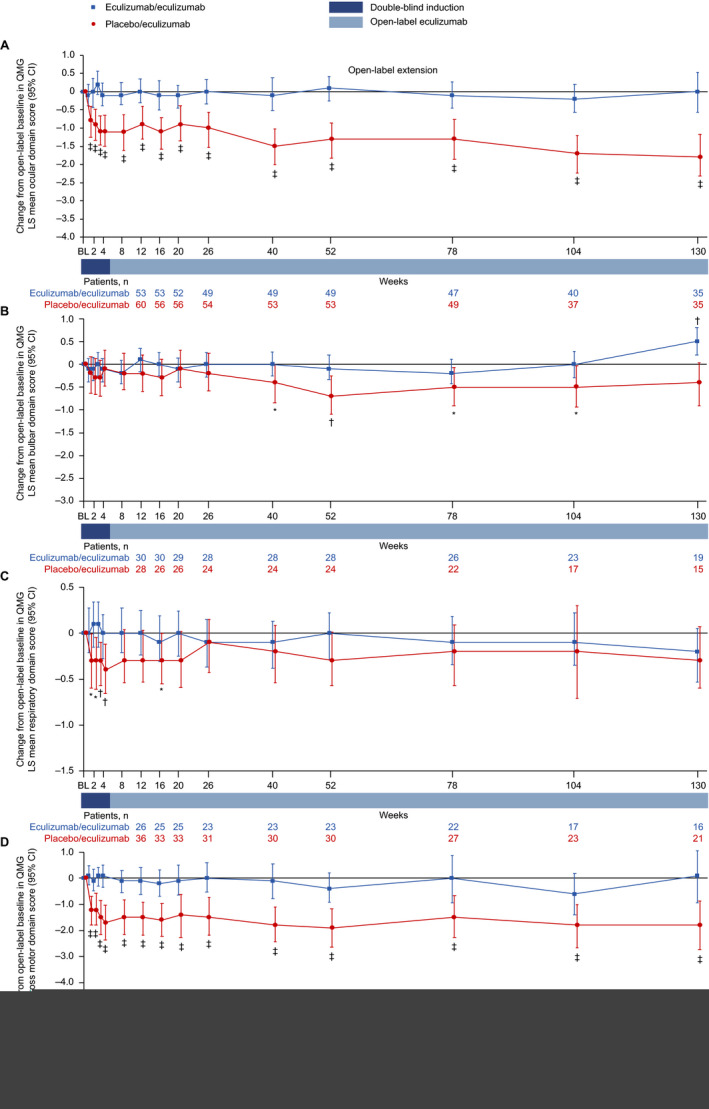
Changes in QMG LS mean domain scores from open‐label baseline to week 130 for (A) ocular, (B) bulbar, (C) respiratory, and (D) gross motor domains. *P ≤ 0.05, ^†^ *P* ≤ 0.01, ^‡^ *P* ≤ 0.001 compared with open‐label baseline, repeated‐measures analysis. Patient numbers were not the same for each assessment. Stable scores in the eculizumab/eculizumab group are evidence of maintained improvement achieved during eculizumab treatment in REGAIN in these patients. Data are from patients with abnormal domain scores at REGAIN baseline only. BL = baseline; CI = confidence interval; LS = least‐squares; QMG = quantitative myasthenia gravis scale.

Patients in the eculizumab/eculizumab group experienced rapid improvements across all four QMG domains during REGAIN, and this treatment effect was sustained through 130 weeks of the OLE (Fig. 7A–D and Fig. 8A–D). During REGAIN, peak improvements were observed by week 26 in the ocular and gross motor domains (Fig. 7A, D), week 20 in the bulbar domain (Fig. 7B), and week 12 in the respiratory domain (Fig. 7C). Thereafter, the peak improvements achieved in REGAIN were sustained or increased in all domains during the OLE through 130 weeks, except in the bulbar domain at week 130 (Fig. 8A–D).

In the placebo/eculizumab group, statistically significant improvements from open‐label baseline were observed across all domains by week 1, except for the bulbar domain, which was first significantly improved at week 40 (Fig. 8A–D). In the ocular and gross motor domains, improvements from open‐label baseline were significant for every week through 130 weeks (Fig. 8A, D). In the bulbar and respiratory domains, improvements from open‐label baseline were significant at weeks 40, 52, 78, and 104, and at weeks 1, 2, 3, 4, and 16, respectively (Fig. 8B, C).

## Discussion

Eculizumab is effective in the long‐term treatment of patients with refractory AChR+ gMG.^19^ In the REGAIN OLE, rapid improvements in MG‐ADL and QMG total scores with eculizumab were observed to be sustained through 156 weeks. Final OLE safety data were consistent with interim OLE safety data and eculizumab’s known safety profile in MG.^15^, ^19^ (Mantegazza R, et al. 2020. Minimal manifestations in eculizumab‐treated patients with refractory myasthenia gravis. Manuscript submitted for publication).

We previously reported that the correlation between MG‐ADL and QMG total scores was stronger when assessing response to treatment than baseline status^23^ and that there was a strong correlation between scores after 26 weeks of eculizumab treatment.^24^ Correlation analysis of the final OLE data confirms these observations for long‐term eculizumab treatment (median treatment duration was over 2.5 years). The strong correlations between MG‐ADL and QMG total scores, both for changes from eculizumab baseline to last OLE assessment and at last OLE assessment, demonstrate that the long‐term effect of eculizumab is consistent between the MG‐ADL and QMG assessments. This finding further validates use of the MG‐ADL in assessing response to treatment in MG and provides evidence that patient‐reported improvements in response to long‐term eculizumab treatment are supported by objective physician assessments of patients with refractory AChR+ gMG.

At REGAIN baseline, all study participants had at least one abnormal domain score for both MG‐ADL and QMG, and had experienced inadequate treatment responses with standard ISTs. In contrast to other MG therapies that have been reported to have differential effects across muscle groups and onsets of action of up to 12 months,^25^, ^26^ improvements were observed across the MG‐ADL domains for all four muscle groups within a few weeks of eculizumab initiation and maintained through at least 130 weeks. This demonstrates that eculizumab elicits rapid and sustained improvements in patients’ breathing and functioning in a wide range of activities of daily living. These patient‐reported improvements were supported by objective, physician‐reported improvements in muscle strength with eculizumab, as evaluated by QMG domain scores across all four muscle groups, which were also rapid and sustained through at least 130 weeks. It remains a possibility that there is a subset of patients who respond to eculizumab later than what is observed in the study population as a whole; this hypothesis is currently being evaluated in a separate analysis (Howard J, et al. Long‐term efficacy of eculizumab in refractory generalized myasthenia gravis: responder analyses. Manuscript in development).

It is notable that these improvements were achieved by patients with refractory disease, a population with significant disease burden,^1^ and that most participants completed the OLE. These data are important in the context of clinical decision‐making for patients with gMG, regardless of whether their symptoms are primarily driven by a single domain (for example, ocular) or by multiple domains. Eculizumab, therefore, alleviates a significant and unmanaged disease burden across different muscle groups that affect quality of life, confirming the efficacy and value of inhibiting the C5 complement protein as a therapeutic strategy for patients with refractory AChR+ gMG.

The OLE of REGAIN allowed all patients who completed REGAIN to receive long‐term eculizumab treatment and, thus, yielded long‐term data on its safety and effectiveness. Limitations of the REGAIN study and its OLE have been discussed previously.^15^, ^19^ The open‐label design of the OLE component of this study is its main limitation, which is a potential source of bias in reporting from both patients and physicians. There were no substantial changes, however, in MG‐ADL and QMG total or domain scores in the eculizumab/eculizumab group from the open‐label baseline, suggesting that any potential open‐label reporting bias was inconsequential in this analysis. In addition, the blinded induction phase at the start of the OLE confirmed the rapid treatment effect of eculizumab in patients who had received placebo during REGAIN.

In summary, the results reported here confirm previous REGAIN and interim OLE data showing that eculizumab treatment results in rapid improvements in MG‐ADL and QMG total scores that are sustained through at least 130 weeks in individuals with refractory AChR+ gMG.^19^ Furthermore, the current analysis demonstrates that the rapid and sustained effects of eculizumab in this patient population are evident across the different MG‐ADL and QMG domains representing ocular, respiratory, bulbar, and limb/gross motor muscle groups. It also demonstrates consistency between patient and physician evaluations. Eculizumab treatment, therefore, rapidly induces clinical benefits across muscle groups, from both patient and physician perspectives, which are maintained in the long term in individuals with refractory AChR+ gMG.

## Author Contributions

R.M., F.L.O'B., M.Y., and J.F.H. contributed to the conception and design of the study, acquisition and analysis of data, and drafting the text and preparing the figures. REGAIN study group members are listed in Supplementary Table S1.

## Conflicts of Interest

R.M. and J.F.H. have received research support, honoraria, and nonfinancial support from Alexion Pharmaceuticals, which owns patent rights to eculizumab that was used in this study. F.L.O'B. and M.Y. are employed by, and own stock in, Alexion Pharmaceuticals, which owns patent rights to eculizumab that was used in this study.

## Supporting information


**Supplementary Table S1**. Complete list of study investigators.

## Data Availability

Alexion will consider requests for disclosure of clinical study participant‐level data provided that participant privacy is assured through methods like data deidentification, pseudonymization, or anonymization (as required by applicable law), and if such disclosure was included in the relevant study informed consent form or similar documentation. Qualified academic investigators may request participant‐level clinical data and supporting documents (statistical analysis plan and protocol) pertaining to Alexion‐sponsored studies. Further details regarding data availability and instructions for requesting information are available in the Alexion Clinical Trials Disclosure and Transparency Policy at http://alexion.com/research‐development. Link to data request form: https://alexion.com/contact‐alexion/medical‐information.

## References

[1] Engel‐Nitz NM , Boscoe AN , Wolbeck R , et al. Burden of illness in patients with treatment refractory myasthenia gravis. Muscle Nerve 2018;58:99–105. 10.1002/mus.26114 29486521

[2] Lindstrom JM , Seybold ME , Lennon VA , et al. Antibody to acetylcholine receptor in myasthenia gravis. Prevalence, clinical correlates, and diagnostic value. Neurology 1976;26:1054–1059. 988512 10.1212/wnl.26.11.1054

[3] Mantegazza R , Pareyson D , Baggi F , et al. Anti AChR antibody: relevance to diagnosis and clinical aspects of myasthenia gravis. Ital J Neurol Sci 1988;9:141–145. 3397267 10.1007/BF02337460

[4] Oh SJ , Kim DE , Kuruoglu R , et al. Diagnostic sensitivity of the laboratory tests in myasthenia gravis. Muscle Nerve 1992;15:720–724. 1324429 10.1002/mus.880150616

[5] Somnier FE . Clinical implementation of anti‐acetylcholine receptor antibodies. J Neurol Neurosurg Psychiatry 1993;56:496–504. 8505642 10.1136/jnnp.56.5.496 PMC1015008

[6] Vincent A , McConville J , Farrugia ME , et al. Antibodies in myasthenia gravis and related disorders. Ann N Y Acad Sci 2003;998:324–335. 14592891 10.1196/annals.1254.036

[7] Vincent A , Newsom‐Davis J . Acetylcholine receptor antibody as a diagnostic test for myasthenia gravis: results in 153 validated cases and 2967 diagnostic assays. J Neurol Neurosurg Psychiatry 1985;48:1246–1252. 4087000 10.1136/jnnp.48.12.1246 PMC1028609

[8] Grob D , Brunner N , Namba T , et al. Lifetime course of myasthenia gravis. Muscle Nerve 2008;37:141–149. 18059039 10.1002/mus.20950

[9] Robertson NP , Deans J , Compston DA . Myasthenia gravis: a population based epidemiological study in Cambridgeshire, England. J Neurol Neurosurg Psychiatry 1998;65:492–496. 9771771 10.1136/jnnp.65.4.492 PMC2170309

[10] Suh J , Goldstein JM , Nowak RJ . Clinical characteristics of refractory myasthenia gravis patients. Yale J Biol Med 2013;86:255–260. 23766745 PMC3670444

[11] Nowak RJ , Dicapua DB , Zebardast N , et al. Response of patients with refractory myasthenia gravis to rituximab: a retrospective study. Ther Adv Neurol Disord 2011;4:259–266. 22010039 10.1177/1756285611411503 PMC3187675

[12] Drachman DB , Adams RN , Hu R , et al. Rebooting the immune system with high‐dose cyclophosphamide for treatment of refractory myasthenia gravis. Ann N Y Acad Sci 2008;1132:305–314. 18567882 10.1196/annals.1405.033 PMC3390145

[13] Silvestri NJ , Wolfe GI . Treatment‐refractory myasthenia gravis. J Clin Neuromuscul Dis 2014;15:167–178. 24872217 10.1097/CND.0000000000000034

[14] Buzzard KA , Meyer NJ , Hardy TA , et al. Induction intravenous cyclophosphamide followed by maintenance oral immunosuppression in refractory myasthenia gravis. Muscle Nerve 2015;52:204–210. 25487528 10.1002/mus.24536

[15] Howard JF Jr , Utsugisawa K , Benatar M , et al. Safety and efficacy of eculizumab in anti‐acetylcholine receptor antibody‐positive refractory generalised myasthenia gravis (REGAIN): a phase 3, randomised, double‐blind, placebo‐controlled, multicentre study. Lancet Neurol 2017;16:976–986. 29066163 10.1016/S1474-4422(17)30369-1

[16] Rother RP , Rollins SA , Mojcik CF , et al. Discovery and development of the complement inhibitor eculizumab for the treatment of paroxysmal nocturnal hemoglobinuria. Nat Biotechnol 2007;25:1256–1264. 17989688 10.1038/nbt1344

[17] Biesecker G , Gomez CM . Inhibition of acute passive transfer experimental autoimmune myasthenia gravis with Fab antibody to complement C6. J Immunol 1989;142:2654–2659. 2703710

[18] Conti‐Fine BM , Milani M , Kaminski HJ . Myasthenia gravis: past, present, and future. J Clin Invest 2006;116:2843–2854. 17080188 10.1172/JCI29894 PMC1626141

[19] Muppidi S , Utsugisawa K , Benatar M , et al. Long‐term safety and efficacy of eculizumab in generalized myasthenia gravis. Muscle Nerve 2019;60:14–24. 30767274 10.1002/mus.26447 PMC6619057

[20] Wolfe GI , Herbelin L , Nations SP , et al. Myasthenia gravis activities of daily living profile. Neurology 1999;52:1487–1489. 10227640 10.1212/wnl.52.7.1487

[21] Barohn RJ , McIntire D , Herbelin L , et al. Reliability testing of the quantitative myasthenia gravis score. Ann N Y Acad Sci 1998;841:769–772. 9668327 10.1111/j.1749-6632.1998.tb11015.x

[22] Campbell MJ , Swinscow TDV . Statistics at square one, 11th ed Hoboken, NJ: Wiley‐Blackwell, 2009.

[23] Howard JF Jr , Freimer M , O'Brien F , et al. QMG and MG‐ADL correlations: study of eculizumab treatment of myasthenia gravis. Muscle Nerve 2017;56:328–330. 28010051 10.1002/mus.25529

[24] Vissing J , O'Brien F , Wang JJ , et al. Correlation between myasthenia gravis‐activities of daily living (MG‐ADL) and quantitative myasthenia gravis (QMG) assessments of anti‐acetylcholine receptor antibody‐positive refractory generalized myasthenia gravis in the phase 3 REGAIN study. Muscle Nerve 2018;58:E21–E22. 29684239 10.1002/mus.26152

[25] Barnett C , Bril V , Kapral M , et al. Myasthenia gravis impairment index: responsiveness, meaningful change, and relative efficiency. Neurology 2017;89:2357–2364. 29101274 10.1212/WNL.0000000000004676 PMC5719924

[26] Guptill JT , Soni M , Meriggioli MN . Current treatment, emerging translational therapies, and new therapeutic targets for autoimmune myasthenia gravis. Neurotherapeutics 2016;13:118–131. 26510558 10.1007/s13311-015-0398-y PMC4720661

