# Supplementary material for: Consistent improvement with eculizumab across muscle groups in myasthenia gravis
Source: Ann Clin Transl Neurol. 2020 Jul 22;7(8):1327–39. doi: 10.1002/acn3.51121 (PMC7448154; doi:10.1002/acn3.51121)
Supplement: Supplementary file 1 — Supplementary Table S1. Complete list of study investigators. [file ACN3-7-1327-s001.docx]

# Supplementary materials

## Supplementary Table 1: complete list of study investigators

| Argentina |
| --- |
| **Instituto de Investigaciones Neurológicas Raúl Carrea (FLENI), Buenos Aires:** Principal investigators: Claudio Gabriel Mazia, Miguel Wilken; sub-investigators: Miguel Wilken, Fabio Barroso; study coordinator: Juliet Saba. |
| **Hospital Italiano de Buenos Aires, Buenos Aires:** Principal investigator: Marcelo Rugiero; sub-investigators: Mariela Bettini, Marcelo Chaves, Gonzalo Vidal; study coordinator: Alejandra Dalila Garcia. |
| **Belgium** |
| **Algemeen Ziekenhuis Sint-Lucas – Campus Sint-Lucas, Ghent**: Principal investigator: Jan De Bleecker; sub-investigator: Guy Van den Abeele; primary study coordinator: Kathy de Koning; study coordinator: Katrien De Mey. |
| **Universitair Ziekenhuis Antwerpen, Edegem, Antwerp:** Principal investigator: Rudy Mercelis; study coordinators: Délphine Mahieu, Linda Wagemaekers. |
| **Universitaire Ziekenhuizen Leuven, Leuven:** Principal investigator: Philip Van Damme (Laboratory of Neurobiology, Department of Neuroscience, Katholieke Universiteit Leuven and Center for Brain & Disease Research); sub-investigators: Annelies Depreitere, Caroline Schotte, Charlotte Smetcoren, Olivier Stevens, Sien Van Daele, Nicolas Vandenbussche, Annelies Vanhee, Sarah Verjans, Jan Vynckier; primary study coordinator: Ann D’Hont; study coordinator: Petra Tilkin. |
| **Brazil** |
| **Faculdade de Medicina do ABC, Santo André:** Principal investigator: Alzira Alves de Siqueira Carvalho; sub-investigators: Igor Dias Brockhausen, David Feder; study coordinators: Daniel Ambrosio, Pamela César, Ana Paula Melo, Renata Martins Ribeiro, Rosana Rocha, Bruno Bezerra Rosa, Thabata Veiga; study coordinators (back-up): Luiz Augusto da Silva, Murilo Santos Engel, Jordana Gonçalves Geraldo. |
| **Fundação Faculdade Regional de Medicina de São José do Rio Preto, São José do Rio Preto:** Principal investigator: Maria da Penha Ananias Morita; sub-investigators: Erica Nogueira Coelho, Gabriel Paiva, Marina Pozo, Natalia Prando; study coordinators: Debora Dada Martineli Torres, Cristiani Fernanda Butinhao, Gustavo Duran, Tomás Augusto Suriane Fialho, Tamires Cristina Gomes da Silva, Luiz Otavio Maia Gonçalves, Lucas Eduardo Pazetto, Luciana Renata Cubas Volpe, Luciana Souza Duca. |
| **Hospital Mãe de Deus, Porto Alegre:** Principal investigator: Maurício André Gheller Friedrich; sub-investigator: Alexandre Guerreiro, Henrique Mohr, Maurer Pereira Martins; study coordinators: Daiane da Cruz Pacheco, Luciana Ferreira, Ana Paula Macagnan, Graziela Pinto, Aline de Cassia Santos. |
| **Universidade Federal de São Paulo [UNIFESP], São Paulo:** Principal investigator: Acary Souza Bulle Oliveira; sub-investigators: Ana Carolina Amaral de Andrade, Marcelo Annes, Liene Duarte Silva, Valeria Cavalcante Lino, Wladimir Pinto; study coordinators: Natália Assis, Fernanda Carrara, Carolina Miranda, Iandra Souza; study coordinator (back-up): Patrícia Fernandes. |
| **Canada** |
| **University of Alberta Hospital, Edmonton, AB:** Principal investigator: Zaeem Siddiqi; sub-investigator: Cecile Phan; primary study coordinator: Jeffrey Narayan; study coordinators; Derrick Blackmore, Ashley Mallon, Rikki Roderus, Elizabeth Watt. |
| **Czech Republic** |
| **Fakultní nemocnice Brno, Brno**: Principal investigator: Stanislav Vohanka; sub-investigators: Josef Bednarik, Magda Chmelikova, Marek Cierny; study coordinator: Stanislava Toncrova. |
| **Fakultní nemocnice Ostrava, Ostrava:** Principal investigator: Jana Junkerova; sub-investigators: Barbora Kurkova, Katarina Reguliova, Olga Zapletalova. |
| **Všeobecná fakultní nemocnice v Praze, Praze:** Principal investigator: Jiri Pitha; sub-investigators: Iveta Novakova, Michaela Tyblova; study coordinators: Ivana Jurajdova, Marcela Wolfova. |
| **Denmark** |
| **Århus Universitetshospital, Aarhus**: Principal investigator: Henning Andersen; sub-investigators: Thomas Harbo, Lotte Vinge; primary study coordinator: Susanne Krogh; study coordinator: Anita Mogensen. |
| **Rigshospitalet, Copenhagen**: Principal investigator: John Vissing; sub-investigators: Joan Højgaard, Nanna Witting; primary study coordinator: Anne Mette Ostergaard Autzen; study coordinator: Jane Pedersen. |
| **Finland** |
| **Neuro NEO Oy, Turku:** Principal investigator: Juha-Pekka Erälinna; sub-investigator: Mikko Laaksonen; Olli Oksaranta; primary study coordinator: Tuula Harrison; study coordinator (back-up): Jaana Eriksson. |
| **Hungary** |
| **Jahn Ferenc Dél-Pesti Kórház, Budapest:** Principal investigator: Csilla Rozsa; sub-investigators: Melinda Horvath, Gabor Lovas, Judit Matolcsi, Gyorgyi Szabo; study coordinators: Gedeonne Jakab, Brigitta Szabadosne. |
| **University of Szeged, Albert Szent-Györgyi Health Center, Szeged:** Principal investigator: Laszlo Vecsei; sub-investigators: Livia Dezsi, Edina Varga; study coordinator: Monika Konyane. |
| **Italy** |
| **Azienda Ospedaliera Sant'Andrea – Università di Roma La Sapienza, Rome**: Principal investigator: Giovanni Antonini; sub-investigators: Antonella Di Pasquale, Matteo Garibaldi, Stefania Morino, Fernanda Troili, Laura Fionda; primary study coordinators: Antonella Di Pasquale, Matteo Garibaldi. |
| **Azienda Ospedaliera Universitaria “Federico II”, Naples:**Principal investigator: Francesco Saccà; previous principal investigator: Alessandro Filla; sub-investigators; Teresa Costabile, Enrico Marano; study coordinators: Angiola Fasanaro, Angela Marsili, Giorgia Puorro. |
| **Fondazione IRCCS Istituto Neurologico Carlo Besta, Milano:** Principal investigator: Renato Mantegazza; sub-investigators: Carlo Antozzi, Silvia Bonanno, Giorgia Camera, Alberta Locatelli, Lorenzo Maggi, Maria Pasanisi; study coordinator: Angela Campanella. |
| **Policlinico Universitario Agostino Gemelli, Rome**: Principal investigator: Amelia Evoli; sub-investigators: Paolo Emilio Alboini, Valentina D’Amato, Raffaele Iorio. |
| **Umberto I Policlinico di Roma – Università di Roma La Sapienza, Rome:** Principal investigator: Maurizio Inghilleri; sub-investigators: Laura Fionda, Vittorio Frasca, Elena Giacomelli, Maria Gori, Diego Lopergolo, Emanuela Onesti; study coordinators: Vittorio Frasca, Maria Gabriele. |
| **Japan** |
| **Chiba University Hospital, Chiba:** Principal investigator: Akiyuki Uzawa; sub-investigators: Tetsuya Kanai, Naoki Kawaguchi, Masahiro Mori; primary study coordinator: Yoko Kaneko; study coordinators: Akiko Kanzaki, Eri Kobayashi. |
| **Kyushu University Hospital, Fukuoka:** Principal investigator: Hiroyuki Murai; sub-investigators: Katsuhisa Masaki, Dai Matsuse, Takuya Matsushita, Taira Uehara; primary study coordinator: Misa Shimpo; study coordinators: Maki Jingu, Keiko Kikutake, Yumiko Nakamura; study coordinator (back-up): Yoshiko Sano. |
| **Hanamaki General Hospital, Hanamaki:** Principal investigator: Kimiaki Utsugisawa; sub-investigator: Yuriko Nagane; primary study coordinators: Ikuko Kamegamori, Tomoko Tsuda; study coordinators: Yuko Fujii, Kazumi Futono, Yukiko Ozawa, Aya Mizugami, Yuka Saito. |
| **Kinki University Hospital, Osaka**: Principal investigator: Makoto Samukawa; previous principal investigator; Hidekazu Suzuki; sub-investigator: Miyuki Morikawa; primary study coordinators: Sachiko Kamakura, Eriko Miyawaki. |
| **Nagasaki University Hospital, Nagasaki:** Principal investigator: Hirokazu Shiraishi; sub-investigators: Teiichiro Mitazaki, Masakatsu Motomura, Akihiro Mukaino, Shunsuke Yoshimura; primary study coordinators: Shizuka Asada, Seiko Yoshida; study coordinators: Shoko Amamoto, Tomomi Kobashikawa, Megumi Koga, Yasuko Maeda, Kazumi Takada, Mihoko Takada, Masako Tsurumaru, Yumi Yamashita, Seiko Yoshida. |
| **National Hospital Organization Sendai Medical Center, Sendai:** Principal investigator: Yasushi Suzuki; sub-investigators: Tetsuya Akiyama, Koichi Narikawa, Ohito Tano, Kenichi Tsukita; primary study coordinators: Rikako Kurihara, Fumie Meguro; study coordinators: Yusuke Fukuda, Miwako Sato. |
| **Osaka University Hospital, Osaka:** Principal investigator: Meinoshin Okumura; sub-investigators: Soichiro Funaka, Tomohiro Kawamura, Masayuki Makamori, Masanori Takahashi; primary study coordinator: Namie Taichi; study coordinators: Tomoya Hasuike, Eriko Higuchi, Hisako Kobayashi, Kaori Osakada. |
| **Sapporo Medical University Hospital, Chuo-ku, Sapporo:** Principal investigators: Tomihiro Imai, Emiko Tsuda; previous principal investigator: Shun Shimohama; sub-investigators: Takashi Hayashi, Shin Hisahara, Tomihiro Imai, Jun Kawamata, Takashi Murahara, Masaki Saitoh, Shun Shimohama, Shuichiro Suzuki, Daisuke Yamamoto; primary study coordinator: Yoko Ishiyama; study coordinators: Naoko Ishiyama, Mayuko Noshiro, Rumi Takeyama, Kaori Uwasa, Ikuko Yasuda. |
| **Republic of Korea** |
| **Korea University Anam Hospital, Seoul:** Principal investigator: Byung-Jo Kim; sub-investigators: Chang Nyoung Lee, Yong Seo Koo, Hung Youl Seok: study coordinators: Hoo Nam Kang, HyeJin Ra. |
| **Samsung Medical Center, Seoul:** Principal investigator: Byoung Joon Kim; sub-investigators: Eun Bin Cho, MiSong Choi, HyeLim Lee, Ju-Hong Min, Jinmyoung Seok; study coordinators: JiEun Lee, Da Yoon Koh, JuYoung Kwon, SangAe Park; study coordinator (back-up): Eun Haw Choi. |
| **Seoul National University Seoul Metropolitan Government Boramae Medical Center, Seoul:**Principal investigator: Yoon-Ho Hong; sub-investigators: So-Hyun Ahn, Dae Lim Koo, Jae-Sung Lim, Chae Won Shin; study coordinators: Ji Ye Hwang, Miri Kim. |
| **Severance Hospital, Yonsei University Health System, Seoul:** Principal investigator: Seung Min Kim; sub-investigators: Ha-Neul Jeong, JinWoo Jung, Yool-hee Kim, Hyung Seok Lee, Ha Young Shin; study coordinators: Eun Bi Hwang, Miju Shin. |
| **Netherlands** |
| **Academisch Medisch Centrum, Amsterdam:** Principal investigator: Anneke van der Kooi; sub-investigator: Marianne de Visser; primary study coordinator: Tamar Gibson. |
| **Spain** |
| **Hospital Universitari de Bellvitge, Barcelona:** Principal investigator: Carlos Casasnovas; sub-investigators: Maria Antonia Alberti Aguilo, Christian Homedes-Pedret, Natalia Julia Palacios, Laura Diez Porras, Valentina Velez Santamaria; primary study coordinator: Ana Lazaro. |
| **Hospital Universitario La Paz, Madrid:** Principal investigator: Exuperio Diez Tejedor, Pilar Gomez Salcedo; sub-investigators: Mireya Fernandez-Fournier, Pedro Lopez Ruiz, Francisco Javier Rodriguez de Rivera; primary study coordinator: Mireya Fernandez-Fournier; study coordinator: Maria Sastre. |
| **Hospital Universitari Vall d'Hebron, Barcelona:** Principal investigator: Josep Gamez Carbonell, Pilar Sune; sub-investigator: Maria Salvado Figueras; primary study coordinator: Gisela Gili, Gonzalo Mazuela. |
| **Hospital Sant Pau, Universitat Autònoma de Barcelona:** Principal investigator: Isabel Illa; sub-investigators: Elena Cortes Vicente, Jordi Diaz-Manera, Luis Antonio Querol Gutiérrez, Ricardo Rojas Garcia; primary study coordinator: Nuria Vidal; study coordinator (back-up): Elisabet Arribas-Ibar. |
| **Sweden** |
| **Karolinska University Hospital, Stockholm:**Principal investigator: Fredrik Piehl; sub-investigator: Albert Hietala; primary study coordinator: Lena Bjarbo. |
| **Turkey** |
| **Dokuz Eylül University Faculty of Medicine, Izmir:** Principal investigator: Ihsan Sengun; sub-investigators: Arzu Meherremova, Pinar Ozcelik; study coordinators: Bengu Balkan, Celal Tuga, Muzeyyen Ugur. |
| **Hacettepe University Faculty of Medicine, Ankara:**Principal investigator: Sevim Erdem-Ozdamar; sub-investigators: Can Ebru Bekircan-Kurt, Nazire Pinar Acar, Ezgi Yilmaz; primary study coordinator: Yagmur Caliskan; study coordinator: Gulsah Orsel. |
| **Kocaeli University Faculty of Medicine, Kocaeli:** Principal investigator: Husnu Efendi; sub-investigators: Seda Aydinlik, Hakan Cavus, Ayse Kutlu; study coordinators: Gulsah Becerikli, Cansu Semiz, Ozlem Tun. |
| **Ondokuz Mayis University Medical Faculty, Atakum/Samsun:** Principal investigator: Murat Terzi; sub-investigators: Baki Dogan, Musa Kazim Onar, Sedat Sen; study coordinators: Tugce Kirbas Cavdar, Adife Veske. |
| **United Kingdom** |
| **King’s College Hospital, London:** Principal investigator: Fiona Norwood; sub-investigators: Aikaterini Dimitriou, Jakit Gollogly, Mohamed Mahdi-Rogers, Arshira Seddigh, Giannis Sokratous; study coordinators: Gal Maier, Faisal Sohail. |
| **Queen Elizabeth Neuroscience Centre, (Wellcome Trust CRF, University Hospitals Birmingham), Birmingham:** Principal investigator: Saiju Jacob; sub-investigators: Girija Sadalage, Pravin Torane; primary study coordinators: Claire Brown, Amna Shah. |
| **The Walton Centre, Liverpool:** Principal investigator: Sivakumar Sathasivam; sub-investigator: Heike Arndt; primary study coordinator: Debbie Davies; study coordinator: Dave Watling. |
| **United States of America** |
| **Brigham and Women's Hospital, Boston, MA:** Principal investigator: Anthony Amato; sub-investigators: Thomas Cochrane, Mohammed Salajegheh; primary study coordinator: Kristen Roe; study coordinators (back-up): Katherine Amato, Shirli Toska. |
| **University at Buffalo Jacobs School of Medicine and Biomedical Sciences, Buffalo, NY:** Principal investigator: Gil Wolfe; sub-investigator: Nicholas Silvestri; study coordinators (back-up): Kara Patrick, Karen Zakalik. |
| **California Pacific Medical Center, San Francisco, CA:** Principal investigator: Jonathan Katz; sub-investigator: Robert Miller; study coordinators: Marguerite Engel, Dallas Forshew. |
| **Carolinas HealthCare System, Charlotte, NC:** Principal investigator: Elena Bravver; sub-investigators: Benjamin Brooks, Mohammed Sanjak; study coordinators: Sarah Plevka, Maryanne Burdette; Scott Cunningham, Mohammad Sanjak; study coordinators (back-up): Megan Kramer, Joanne Nemeth, Clara Schommer (regulator coordinator)**,** Scott Tinerney. |
| **Duke University Health System, Durham, NC:** Principal investigator: Vern Juel; sub-investigators: Jeffrey Guptill, Lisa Hobson-Webb, Janice Massey; primary study coordinators: Kate Beck, Donna Carnes; study coordinator: John Loor; study coordinator (back-up): Amanda Anderson. |
| **Indiana University, Indianapolis, IN**: Principal investigator: Robert Pascuzzi; Sub-investigators: Cynthia Bodkin, John Kincaid, Riley Snook; primary study coordinator: Sandra Guinrich; study coordinator (back-up): Angela Micheels. |
| **Johns Hopkins University School of Medicine, Baltimore, MD**: Principal investigator: Vinay Chaudhry; sub-investigator: Andrea Corse; primary study coordinator: Betsy Mosmiller; study coordinator (back-up): Andrea Kelley. |
| **Lahey Hospital and Medical Center – Burlington, Burlington, MA:** Principal investigator: Doreen Ho; sub-investigators: Jayashri Srinivasan, Michael Vytopil; primary study coordinators: Jordan Jara, Nicholas Ventura; study coordinators: Cynthia Carter, Craig Donahue, Carol Herbert, Stephanie Scala, Elaine Weiner; study coordinator (back-up): Sharmeen Alam. |
| **Las Vegas Clinic, Las Vegas, NV**: Principal investigator: Jonathan McKinnon; sub-investigators: Laura Haar, Naya McKinnon; study coordinators: Karan Alcon, Kaitlyn McKenna, Nadia Sattar; study coordinators (back-up): Kevin Daniels, Dennis Jeffery. |
| **Ohio State University Wexner Medical Center, Columbus, OH**: Principal investigator: Miriam Freimer; sub-investigators: Joseph Chad Hoyle, John Kissel; study coordinators: Julie Agriesti, Sharon Chelnick, Louisa Mezache, Colleen Pineda; study coordinator (back-up): Filiz Muharrem. |
| **Oregon Health and Science University, Portland, OR:** Principal investigators: Chafic Karam, Julie Khoury; previous principal investigator: Tessa Marburger; sub-investigator: Harpreet Kaur; primary study coordinator: Diana Dimitrova. |
| **Southern Illinois University School of Medicine, Springfield, IL:** Principal investigator: James Gilchrist; sub-investigator: Brajesh Agrawal, Mona Elsayed; primary study coordinator: Stephanie Kohlrus; study coordinators: Angela Andoin, Taylor Darnell; study coordinators (back-up): Laura Golden, Barbara Lokaitis, Jenna Seelbach. |
| **Stanford University School of Medicine, Stanford, CA:** Principal investigator: Srikanth Muppidi; sub-investigators: Neelam Goyal, Sarada Sakamuri, Yuen T So; study coordinators: Shirley Paulose, Sabrina Pol, Lesly Welsh. |
| **The University of Texas Health Science Center at San Antonio, San Antonio, TX:** Principal investigator: Ratna Bhavaraju-Sanka; sub-investigator: Alejandro Tobon Gonzalez; study coordinators: Lorraine Dishman, Floyd Jones; study coordinators (back-up): Anna Gonzalez, Patricia Padilla, Amy Saklad, Marcela Silva. |
| **The University of Texas Southwestern Medical Center, Dallas, TX**: Principal investigator: Sharon Nations; sub-investigator: Jaya Trivedi; study coordinator: Steve Hopkins. |
| **University of Alabama at Birmingham** **Medicine, Birmingham, AL:** Principal investigator: Mohamed Kazamel; previous principal investigator: Mohammad Alsharabati; sub-investigator: Liang Lu, Kenkichi Nozaki; study coordinator: Sandi Mumfrey-Thomas; study coordinator (back-up): Amy Woodall. |
| **University of California-Irvine, Irvine, CA:** Principal investigator: Tahseen Mozaffar; sub-investigators: Tiyonnoh Cash, Namita Goyal, Gulmohor Roy; study coordinator: Veena Mathew; study coordinators (back-up): Fatima Maqsood, Brian Minton. |
| **University of California-San Francisco-Fresno, CA:** Principal investigator: H. James Jones; previous principal investigator: Jeffrey Rosenfeld; study coordinator: Rebekah Garcia; study coordinators (back-up): Laura Echevarria, Sonia Garcia. |
| **University of Florida Health Jacksonville, Jacksonville, FL:** Principal investigator: Michael Pulley; sub-investigators: Shachie Aranke, Alan Ross Berger, Jaimin Shah; primary study coordinators: Yasmeen Shabbir, Lisa Smith; study coordinator: Mary Varghese; study coordinator (back up): Yasmeen Shabbir. |
| **University of Iowa Children's Hospital, Iowa City, IA**: Principal investigator: Laurie Gutmann; sub-investigators: Ludwig Gutmann, Nivedita Jerath, Christopher Nance, Andrea Swenson; primary study coordinator: Heena Olalde; study coordinator: Nicole Kressin; study coordinator (back-up): Jeri Sieren. |
| **University of Kansas Medical Center, Kansas City, KS:** Principal investigator: Richard Barohn; sub-investigators: Mazen Dimachkie, Melanie Glenn, April McVey, Mamatha Pasnoor, Jeffery Statland, Yunxia Wang; primary study coordinator: Tina Liu; study coordinators: Kelley Emmons, Nicole Jenci, Jerry Locheke; study coordinators (back-up): Alex Fondaw, Kathryn Johns, Gabrielle Rico, Maureen Walsh; lead evaluator trainer: Laura Herbelin. |
| **University of Maryland Medical Center, Baltimore, MD**: Principal investigator: Charlene Hafer-Macko; sub-investigators: Justin Kwan, Lindsay Zilliox; primary study coordinators: Karen Callison, Valerie Young; study coordinator: Beth DiSanzo; study coordinator (back-up): Kerry Naunton. |
| **University of Miami Miller School of Medicine, Miami, FL:** Principal investigator: Michael Benatar; sub-investigators: Martin Bilsker, Khema Sharma; primary study coordinators: Anne Cooley, Eliana Reyes; study coordinators: Sara-Claude Michon, Danielle Sheldon; study coordinator (back-up): Julie Steele. |
| **University of North Carolina Medical Center, Chapel Hill, NC**: Principal investigator: James Howard, Jr; sub-investigator: Chafic Karam, Rebecca Traub; study coordinator: Manisha Chopra. |
| **University of South Florida, Tampa, FL:** Principal investigator: Tuan Vu; sub-investigators: Lara Katzin, Terry McClain; study coordinator: Brittany Harvey; study coordinators (back-up): Adam Hart, Kristin Huynh. |
| **University of Southern California, Los Angeles, CA**: Principal investigator: Said Beydoun; sub-investigators: Amaiak Chilingaryan, Victor Doan, Brian Droker, Hui Gong, Sanaz Karimi, Frank Lin, Terry McClain, Krishna Polaka, Akshay Shah, Anh Tran; study coordinator: Salma Akhter; study coordinator (back-up): Ali Malekniazi. |
| **University of Vermont Medical Center, Burlington, VT:** Principal investigator: Rup Tandan; sub-investigator: Michael Hehir, Waqar Waheed; primary study coordinator: Shannon Lucy. |
| **University of Washington, Seattle, WA**: Principal investigator: Michael Weiss; sub-investigator: Jane Distad; primary study coordinator: Susan Strom; study coordinators: Sharon Downing, Bryan Kim. |
| **Wesley Neurology Clinic, PC, Cordova, TN:** Principal investigator: Tulio Bertorini; sub-investigators: Thomas Arnold, Kendrick Henderson, Rekha Pillai; primary study coordinator: Ye Liu; study coordinator: Lauren Wheeler; study coordinators (back-up): Jasmine Hewlett, Mollie Vanderhook. |
| **Yale University, New Haven, CT:** Principal investigator: Richard Nowak; sub-investigators: Daniel Dicapua, Benison Keung, Aditya Kumar, Huned Patwa, Kimberly Robeson, Irene Yang; study coordinator: Joan Nye; study coordinator (back-up): Hong Vu |
